# Exploring the hypothetical role of cerebellar pain prediction errors in fibromyalgia-associated chronic pain

**DOI:** 10.3389/fneur.2026.1734010

**Published:** 2026-02-09

**Authors:** Emma Pepe, Davide Spinetti, Chiara Ceolin, Roberta Ramonda, Sara Bindoli, Paolo Sfriso, Gabriella Paparella, Michela Sarlo, Giuseppe Sergi, Daniela Mapelli, Marina De Rui, Maria Devita

**Affiliations:** 1 Department of General Psychology (DPG), University of Padua, Padua, Italy; 2 Geriatrics Unit, Department of Medicine (DIMED), University of Padua, Padua, Italy; 3 Rheumatology Unit, Department of Medicine DIMED, University of Padova, Padua, Italy; 4 Scientific Institute IRCCS E. Medea, Treviso, Italy; 5 Department of Communication Sciences, Humanities and International Studies, University of Urbino Carlo Bo, Urbino, Italy

**Keywords:** cerebellum, chronic pain, fibro-fog, fibromyalgia, maladaptive plasticity, prediction error

## Abstract

Despite growing evidence that the cerebellum contributes to sensory, motor, cognitive, and affective domains, its role in chronic pain remains poorly understood. Fibromyalgia (FM), a rheumatological condition in which chronic pain is a hallmark feature, offers a paradigmatic model. Although neuroimaging studies have reported increased cerebellar activity in response to nociceptive stimuli, its contribution to pain chronification has been largely overlooked. This perspective paper proposes that the cerebellum may play a central role in FM by generating persistent prediction errors. Dysregulation of this mechanism may result in a mismatch between sensorimotor inputs and expected outcomes, for both noxious and innocuous stimuli, progressively disrupting error-based learning. We term this hypothesized state ‘cerebellar fragility’, where the system becomes locked into maladaptive loops. Reconceptualizing cerebellar involvement in chronic pain opens new perspectives for research and therapeutic strategies.

## Introduction

Pain is a fundamental homeostatic drive, guiding behavior in the presence of actual or potential tissue damage. Its biological aim is to warn the individual and promote adaptive survival strategies—either avoidance or defensive behaviors such as fight or flight (1). While acute pain is thus adaptive, chronic pain—defined as pain persisting for at least 3 months—loses this protective function and becomes maladaptive, profoundly impacting emotional state, cognitive performance, and quality of life (2). Prolonged nociceptive input induces maladaptive plastic changes along the pain pathways, resulting in central sensitization. This process involves the increased release of excitatory mediators and neurotrophic factors that sensitize peripheral and central nociceptive circuits, sustaining hyperexcitability (3, 4). Although direct evidence remains limited, central sensitization is widely considered the leading hypothesis for the pathophysiology of chronic pain conditions, including fibromyalgia (5).

Fibromyalgia (FM) is a chronic pain syndrome characterized by widespread musculoskeletal pain, fatigue, non-restorative sleep, and cognitive impairments, labeled as “*fibro-fog*,” which impair daily functioning and quality of life (6). Prevalence estimates range from 0.2 to 6.6% worldwide, with a predominance in women (7, 8). Since 1990, diagnostic criteria have evolved considering 11 tender points to multidimensional assessments incorporating pain distribution, fatigue, sleep, and cognitive symptoms using Widespread Pain Index (WPI) and Symptoms Severity Scale (SSS; 58, 59). Neuroimaging studies have traditionally focused on abnormalities in cortical pain networks, identifying reduced gray matter in the cingulate, insula, and prefrontal cortices, as well as altered connectivity within salience and default mode networks (9, 62). However, growing attention has recently shifted to the cerebellum. Traditionally considered as a motor hub, the cerebellum is now recognized as integral to pain modulation due to its connections with thalamic, limbic, and brainstem structures involved in salience attribution and descending inhibition (10). In FM, structural and functional cerebellar abnormalities—including lobular atrophy and hypertrophy, disrupted peduncular integrity, and altered connectivity with prefrontal and temporal cortices—correlate with enhanced pain sensitivity, affective symptoms, and cognitive dysfunction (11–14). However, given these assumptions, the knowledge about the role of the cerebellum in the pathogenesis of FM is still underexplored. We propose a novel hypothesis according to which the cerebellum may contribute to the chronification of pain in FM through the generation of persistent prediction errors. We speculate that a dysregulation in predictive mechanisms may result in a chronic mismatch between sensorimotor inputs and expected outcomes, progressively disrupting error-based learning and driving maladaptive plasticity. Positioning the cerebellum as a central player in the perpetuation of these predictive mismatches, we aim to provide a new framework for understanding the pathophysiology of FM and open new avenues for targeted, cerebellum-focused interventions.

## Cerebral and cerebellum pain pathways

Pain perception arises from the integration of sensory, cognitive, and emotional processes across distributed neural networks (15). Peripheral noxious inputs are detected by Transient Receptor Potential (TRP)-channel–expressing nociceptors, whose pseudounipolar neurons project from dorsal root ganglia to the spinal dorsal horn (4, 16, 17). C and Aδ fibers carry mechanical nociceptive stimuli and terminate mainly in laminae I–II (4). Second-order neurons decussate through the anterior white commissure and ascend within the anterolateral system—principally the spinothalamic tract, with additional contributions from spinoreticular and spinoparabrachial pathways (17, 18). These ascending fibers project to the parabrachial nucleus, which communicates with hypothalamus and amygdala to coordinate autonomic and affective responses, and to thalamic nuclei, specifically, the ventral-postero lateral (VPL), ventral-posteromedial (VPM), and mediodorsal (MD) nuclei (19). These nuclei relay nociceptive information to cortical regions (4, 20). The primary and secondary somatosensory cortices encode spatial, temporal, and qualitative features of pain, while the insula, anterior cingulate cortex (ACC), and prefrontal cortex (PFC) integrate affective, anticipatory, and evaluative dimensions (17, 19). Neuroimaging consistently confirms that painful stimulation activates not only sensory but also limbic and prefrontal areas, underscoring the emotional and cognitive components of pain (21, 22). Descending modulation is mediated through cortical–limbic projections to the periaqueductal gray (PAG), which contains enkephalinergic neurons and projects to the rostroventromedial medulla (RVM) and dorsal horn (3, 23). Within the RVM, ON and OFF cells exert bidirectional control over nociceptive transmission: ON cells facilitate pain, whereas OFF cells inhibit it. Serotonin and noradrenaline play dual roles depending on receptor subtype—antinociceptive via 5-HT₁A/₁B/₁D/₇ and *α*₂, pronociceptive via 5-HT₂A/₃ and α₁ (24–28). This bidirectional balance allows pain to be dynamically shaped by context, emotion, and past experience (29, 30, 60). Thus, pain emerges not as a mere sensory signal but as a multidimensional experience integrating somatosensory, limbic, and cognitive systems. A deeper understanding of the neuroanatomy and neurobiology of pain is not merely a descriptive exercise but rather provides the foundation for broadening our focus toward structures that have been traditionally overlooked. Among these, the cerebellum, long regarded almost exclusively as a center for motor control, has recently emerged as a potential node within pain pathways. As will be detailed in this paper, it maintains both direct and indirect connections with key nociceptive regions and exhibits specific receptor architectures capable of interacting with neurotransmitters involved in pain processing (31). Nociceptive inputs reach the cerebellar cortex via climbing and mossy fibers, spinocerebellar tracts from the inferior olive, and cortico-pontine pathways carrying higher-order cognitive–emotional signals (10, 32). On the efferent side, deep cerebellar nuclei project to thalamic regions (VL, MD, and centromedial–parafascicular complex-CM-Pf), which connect with somatomotor, prefrontal, cingulate, and insular cortices, as well as with PAG, raphe nuclei, and reticular formation—thereby influencing descending pain control (10, 32). Through thalamic relays, these outputs further engage limbic structures such as the amygdala and hippocampus, shaping the affective and contextual aspects of pain (33). Animal studies confirm this bidirectional role: cerebellar stimulation elevates nociceptive thresholds (34), morphine injections into anterior cortex induce analgesia (35), while fastigial modulation reduces visceral reflexes (32). At the level of neural circuit, cerebellar projections to PAG, RVM, and intralaminar thalamus have been identified as key pathways involved in descending pain modulation (10). Human neuroimaging studies support these findings, consistently showing activation of lobules IV–VI and Crus I during cutaneous, muscular, and visceral pain, as well as during pain anticipation (36). Taken together, these data establish the cerebellum as a central hub linking ascending and descending nociceptive pathways, integrating sensory, cognitive, and affective dimensions of pain.

## Cerebellar involvement in fibromyalgia: insight from physiology and neuroimaging

The cerebellum is increasingly recognized as an active participant in nociceptive processing rather than a simple relay. Cortical cerebellar regions can enhance pain salience through connections with prefrontal, insular, and cingulate cortices, whereas fastigial projections to the periaqueductal gray (PAG) and brainstem contribute to descending inhibitory control (36, 37). Functionally, the cerebellum implements internal forward models to predict the sensory consequences of motor commands. Discrepancies between expected and actual input are signaled via climbing fiber–evoked complex spikes, which act as error signals to update internal representations (38–40). Predictive paradigms indicate cerebellar activity during pain anticipation, suggesting that it may generate expectations about upcoming aversive events (1, 10). Over time, this predictive function may support the encoding of intensity, spatial localization, and temporal dynamics of nociceptive stimuli, contributing to the sensory–discriminative dimension of pain (41–43). From a neuroimaging point of view, voxel-based morphometry studies in FM consistently report gray matter reductions in cerebellar lobules IV–V, which correlate with disease duration (44), as well as in lobules VI and VIII (45). Conversely, increases in gray matter volume have been observed in posterior regions such as Crus II and VIIb (46–48). This pattern, characterized by atrophy in sensorimotor lobules versus hypertrophy in cognitive–affective lobules, may reflect stage-dependent pathophysiology or compensatory mechanisms (44, 45, 48–50). Diffusion tensor imaging further indicates altered microstructural integrity in the cerebellar peduncles and other white matter tracts, suggesting disrupted output (12, 13, 47). Functional neuroimaging converges with structural findings. Patients with FM show cerebellar hyperactivation during nociceptive stimulation and at rest (11), involving sensorimotor lobules VI–VIII and cognitive–affective regions such as Crus I/II and VIIb (36). Task-based fMRI shows exaggerated responses to painful stimuli, while resting-state fMRI reveals altered connectivity with prefrontal, insular, and limbic regions (11). Abnormal cerebellar activity is observed both at pain onset and offset, suggesting prolonged modulation of nociceptive processing (11).

### Network connectivity and systemic integration

FM engages a distributed pain network including S1, S2, prefrontal cortex, ACC, insula, thalamus, amygdala, cerebellum, and mesolimbic dopaminergic structures such as the ventral tegmental area (VTA) and ventral striatum (21, 51). As described for the first time by Melzack and Casey (52), the human pain experience can be divided into three dimensions: sensory-discriminative, affective-motivational and cognitive-evaluative (15). It has been suggested that S1 and S2 encode sensory aspects of noxious stimuli, while prefrontal and limbic regions contribute to two dimensions of human pain experience: affective-motivational and cognitive-evaluative (24). Dopaminergic projections from the VTA modulate higher-order processing and interact with descending pathways (3, 53). Connectivity analyses indicate atypical interactions between cerebellar lobules IV–VI, Crus I, and the vermis with medial prefrontal, orbitofrontal, and temporal cortices (5, 12–14). These changes correlate with depressive symptoms, cognitive impairment, and increased sensitivity to experimental pain (5, 12–14). Overall, structural, functional, and connectivity evidence supports a view of the cerebellum as a hub integrating sensory, cognitive, and affective aspects of chronic pain, rather than a passive relay.

## The potential role of the cerebellum in fibromyalgia: from prediction error dysfunction to cerebellar fragility

**Figure 1:**
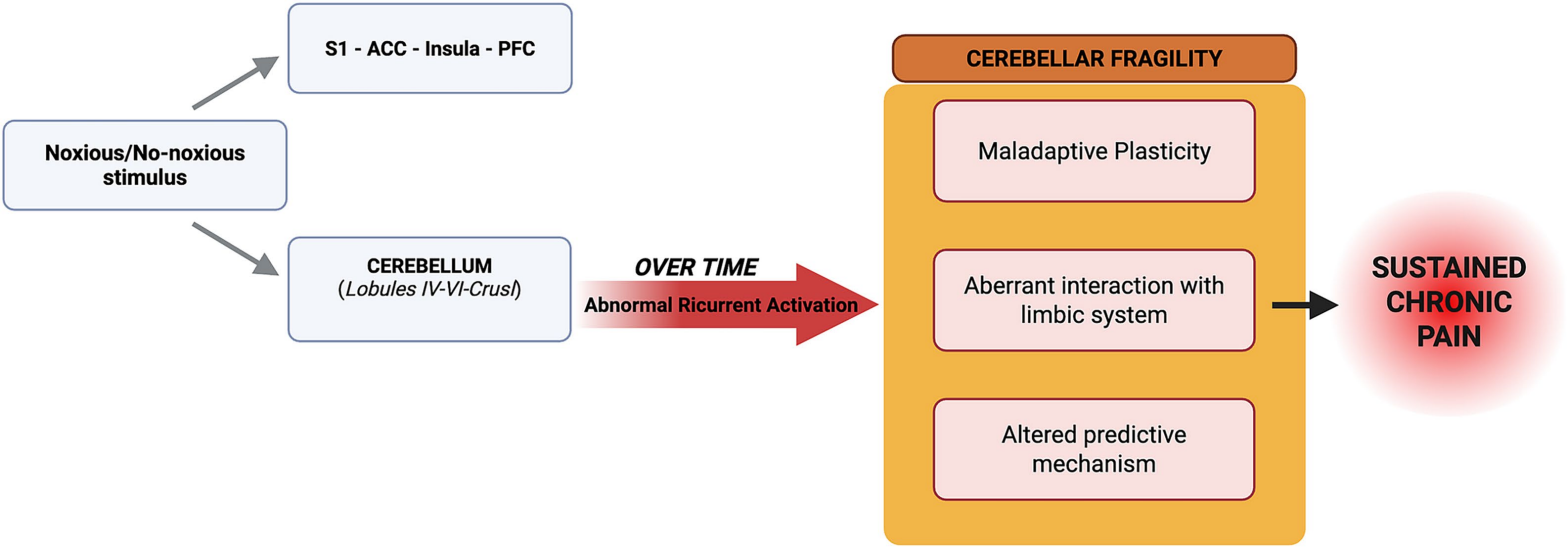
The schematic illustrates the proposed pathway linking prediction error dysfunction to pain chronification. (Left) Noxious and non-noxious stimuli project in parallel to the cortical pain network (S1, ACC, insula, PFC) and the cerebellum (specifically sensorimotor lobules IV–VI and cognitive Crus I). (Center) In the hypothesized FM model, persistent discrepancies between sensory inputs and cortical expectations may generate continuous prediction errors. Over time, this is suggested to result in abnormal recurrent activation, shifting the system into a functional state termed “cerebellar fragility.” (Right) This state, characterized by maladaptive plasticity, aberrant limbic interactions, and altered predictive mechanisms, is proposed to reinforce the maintenance of chronic pain. S1, Primary somatosensory cortex; ACC, Anterior cingulate cortex; PFC, Prefrontal cortex. Created in BioRender. Pepe, E. https://BioRender.com/zkyv541.

Building on converging structural, functional, and connectivity evidence, we propose that cerebellar fragility in fibromyalgia may represent a central mechanism contributing to the persistence of chronic pain. In this context, we could define cerebellar fragility as a growing and progressive vulnerability of cerebellar networks emerging from structural, functional and connectivity alterations, potentially leading to impaired modulatory control over pain-related processes. The cerebellum, through its extensive sensorimotor and cognitive-affective connections, appears well positioned to integrate sensory input, prediction-related signals, and top-down modulatory influences. In FM, the recurrent hyperactivation observed in lobules VI–VIII and Crus I/II, together with region-specific structural alterations such as posterior hypertrophy (e.g., Crus II, vermal VIIb), suggests a shift from an initially compensatory pattern of recruitment to a more enduring and potentially maladaptive reorganization. This multidimensional involvement may render the cerebellum particularly susceptible to dysfunctional plasticity when exposed to persistent nociceptive and non-nociceptive inputs. A speculative yet plausible mechanism involves alterations in predictive processing. Under typical conditions, the cerebellum contributes to building and refining internal models that support the discrimination of sensory events and the estimation of their relevance. In FM, unresolved discrepancies between expected and perceived bodily states could foster a state of persistent error signaling, in which even ambiguous or normally innocuous sensations acquire heightened salience. Abnormal cerebellar error signaling, coupled with maladaptive neuroplasticity and altered communication with prefrontal, insular, and cingulate regions, may contribute to the amplification of sensory, cognitive, and emotional dimensions of pain. Importantly, cerebellar alterations are unlikely to operate in isolation. Aberrant interactions with limbic regions, including the amygdala and hippocampus, as well as prefrontal areas and mesolimbic dopaminergic circuits, point to a broader network architecture in which pain, negative expectations, uncertainty, anxiety, and cognitive biases may become mutually reinforcing. Within this framework, “cerebellar fragility” need not refer solely to anatomical vulnerability, but may instead describe a functional state in which the system struggles to recalibrate perception when faced with noisy or ambiguous inputs. This perspective may help reconcile the diverse symptoms of fibromyalgia as different expressions of a shared mechanism involving dysregulated prediction, impaired error correction, and altered salience processing. Although further empirical validation is required, this framework highlights promising directions for future research and raises the possibility that interventions aimed at stabilizing predictive processes or modulating cerebellar function may offer new therapeutic opportunities (Figure 1).

## Future directions and clinical implications

To evaluate this innovative proposal, it is essential to outline methodological approaches capable of testing its predictions. A key priority is resolving the apparent discrepancy between reports of cerebellar hypertrophy and atrophy (Section 2). Longitudinal neuroimaging studies following individuals with FM from early diagnosis into chronic stages would help clarify whether early volume increases represent compensatory enlargement that later transitions into structural decline, as suggested by cross-sectional evidence (44, 46). Experimental work is equally essential to investigate the proposed dysfunction in predictive coding. A promising starting point draws from recent studies demonstrating the cerebellum’s predictive role in pain processing, such as the increased activity observed in lobule VI and Crus I during pain anticipation (36). We propose adapting “violation-of-expectation” paradigms, widely used in motor learning (40) and associative learning, to the study of nociceptive processing. To rigorously test this, future studies could employ a design including three groups: individuals with fibromyalgia, a clinical control group with typical comorbidities (e.g., anxiety or depression), and healthy controls. This inclusion is critical to control for potential confounding factors, such as anxiety, which can itself elicit sensitivity to somatosensory stimuli (61). Furthermore, if the cerebellum contributes to maladaptive prediction-error signaling, it represents a rational target for therapeutic modulation. Non-invasive brain stimulation (NIBS) techniques, such as transcranial magnetic stimulation (TMS) and transcranial direct current stimulation (tDCS), provide feasible tools to adjust cerebellar excitability. We propose that inhibitory protocols (e.g., cathodal tDCS or low-frequency rTMS) applied to the posterior cerebellum (Crus II/VIIb) may reduce the “noise” generated by persistent error signals (54–57). Finally, early evidence indicates that cerebellar tDCS can alter pain thresholds, but targeted clinical trials are needed to determine whether NIBS can effectively “recalibrate” predictive coding processes in FM.

## Limitations and strengths

This perspective opens new avenues for research and highlights the cerebellum as a promising therapeutic target of growing importance, through both pharmacological and non-pharmacological approaches. However, several limitations of the current literature warrant consideration. Most available findings are derived from cross-sectional studies, restricting the ability to draw causal inferences about the temporal evolution of cerebellar alterations in FM. Moreover, the marked heterogeneity within FM cohorts, including variability in pharmacological treatments, sleep disturbances, and psychiatric comorbidities, introduces confounding factors that future research must systematically address. Despite these limitations, the main strength of this paper is to suggest a new way to reframe the cerebellum in the FM through the lens of “cerebellar fragility” provides a meaningful conceptual advance. This hypothesis moves beyond a strictly corticocentric interpretation and suggests that the persistence of central sensitization may arise from disruptions in the brain’s predictive machinery. Again, in this way the cerebellum takes on a leading role in the context of chronic pain. By targeting what may be conceptualized as a form of “nociceptive dysmetria,” new opportunities may emerge for developing personalized interventions capable of interrupting the chronic pain cycle.

## Conclusion

This perspective article highlights a potential role of the cerebellum in fibromyalgia syndrome. It proposes that the cerebellum undergoes several changes, especially in its functions, such as prediction or expectation and error-based learning. We suggest that these disrupted abilities may exert a detrimental effect, resulting in maladaptive loops that contribute to the persistence of chronic pain over time.

## Data Availability

The original contributions presented in the study are included in the article/supplementary material, further inquiries can be directed to the corresponding author.
